# Evaluation of the expression profile of mRNAs and lncRNAs in cumulus cells associated with polycystic ovary syndrome and pregnancy

**DOI:** 10.22038/IJBMS.2023.69829.15206

**Published:** 2023

**Authors:** Behnaz Hammami, Fatemeh Sadat Mostafavi, Ali Akbari, Seyyed Reza Mousavi, Mohammad Kazemi

**Affiliations:** 1 Department of Genetics and Molecular Biology, School of Medicine, Isfahan University of Medical Science, Isfahan, Iran; 2 Reproductive Sciences and Sexual Health Research Center, Isfahan University of Medical Science, Isfahan, Iran; 3 Department of Anatomical Sciences, School of Medicine, Isfahan University of Medical Science, Isfahan, Iran; 4 Cellular, Molecular and Genetics Research Center, Isfahan University of Medical Sciences, Isfahan, Iran

**Keywords:** Cumulus cells, Infertility, Long non-coding RNA, Polycystic ovary syndrome, Systems biology

## Abstract

**Objective(s):**

Polycystic ovary syndrome (PCOS), the primary cause of anovulatory infertility in women, may change the gene expression profile of cumulus cells. In human ART (assisted reproductive technology), gene expression profiling in cumulus cells, a non-invasive method, may be used to identify the most competent oocytes. We aim to identify key genes according to the network-based data and assess the suitability of these genes as markers to predict oocyte competence and PCOS diagnosis.

**Materials and Methods:**

The GSE34526 microarray dataset was obtained from the Gene Expression Omnibus (GEO) database. The function and pathway enrichment analysis for DEGs were analyzed. A protein-protein interaction (PPI) network analysis and candidate gene screening were conducted. A two-layer network consisting of mRNA and lncRNA was constructed. Expression levels of hub genes were verified using quantitative RT-PCR (qRT-PCR).

**Results:**

A total of 2721 DEGs were retained. The PPI network of selected genes associated with the biological process of “cell communication” was analyzed, and the first 10 key genes were determined by degree. Additionally, 2 hub genes and 2 hub lncRNAs, including *STAT3*, *RHOA*, *GAS5*, and *LINC01116*, were selected from the lncRNA-mRNA network. Finally, expression levels of *STAT3*, *RHOA*, *GAS5*, and *LINC01116* were significantly increased in the cumulus cells of PCOS patients compared to the control group (*P*<0.05). However, there was no significant difference in expression between the pregnant and non-pregnant groups.

**Conclusion:**

*STAT3*, *RHOA*, *GAS5*, and *LINC01116* may serve as possible diagnostic markers for PCOS. However, further studies on a larger population are needed to validate this finding.

## Introduction

Infertility is defined as the failure to achieve clinical pregnancy after 12 months of regular and unprotected sexual intercourse. It is estimated that between 8–12% of reproductive-aged couples in the world are affected (1). As infertility expands worldwide, the importance of ART registries is crucial (2). The choice of high-quality embryos for transfer is largely dependent on the oocyte quality acquired during the maturation process (3).

Examining the morphology of the oocyte, embryo, and cumulus cells is the primary method for choosing the embryos to transfer (4). These morphological and microscopic criteria are subjective, insufficiently precise, and therefore poorly correlated with a successful pregnancy (5). Clinicians have resorted to transferring more than one embryo to obtain higher pregnancy rates, leading to a high incidence of multiple births (6).  Multiple-gestation pregnancy presents risks for both women and infants (7).

 Elective single embryo transfer (eSET), the voluntary transfer of a single high-quality embryo, has considerably reduced multiple gestation rates (8). Due to the limitations of morphologic embryo evaluation, several researchers have turned to supplementary technologies to determine a given embryo’s reproductive potential (9).

Cumulus cells (CCs) are defined as a group of closely related granulosa cells that surround the oocyte and play a role in the maturation and fertilization of the oocyte (10). The interaction between these two cells is bidirectional and occurs through gap junctions and paracrine signals (11, 12). The expression of some genes in cumulus cells has been reported as a potential biomarker of oocyte quality and pregnancy outcome (13).

Polycystic ovary syndrome (PCOS) is the most frequent endocrinopathy affecting reproductive-aged women and the primary cause of anovulatory infertility in women (14). It affects 5–15% of females worldwide (15). This condition is caused by an imbalance of female sex hormones and leads to cysts in the antral follicles of the ovary (16). Although the etiology of the syndrome is not yet fully understood, PCOS is considered a multifactorial disorder with diverse genetic and environmental abnormalities (17). The identification of differentially expressed genes and abnormal pathways in PCOS ovaries may contribute to the understanding of the pathogenesis of PCOS and may lead to the discovery of new treatment approaches (18).

Long noncoding RNAs (LncRNAs) may influence important processes involved in human oocyte maturation, fertilization, and embryonic development, suggesting that they could be valuable biomarkers (19). lncRNAs have pivotal roles in disease development and progression, including endocrine disorders (20). 

Systems biology will assist in the understanding and simplification of biological systems as well as the datasets generated by the high-throughput (HT) technique (21). Also, it has been demonstrated that network-based data could offer an integrated view of the genes or proteins in the network, allowing for a better understanding of the molecular mechanisms underlying the phenotypes of interest (22).

Recently, some research has been carried out to profile cumulus gene expression, identify gene markers, and predict oocyte or embryonic competence (22). However, a two-layer network containing mRNA and lncRNA involved in PCOS and associated with transcript profile GSE34526 has not yet been plotted. There are also a few studies comparing the expression of candidate genes in PCOS and healthy people and their relevance to fertilization and pregnancy.

In this study, we downloaded the microarray dataset GSE34526 from the Gene Expression Omnibus (GEO) database. Subsequently, the function and pathway enrichment analysis for DEGs were analyzed. Additionally, we established a protein-protein interaction (PPI) network, picked out candidate genes, and obtained gene-related lncRNAs. A two-layer network consisting of mRNA and lncRNA was constructed. Expression levels of hub genes were finally verified by the qRT-PCR method. Therefore, according to the network-based data and in order to investigate the correlation between PCOS and the expression of candidate genes, we decided to compare the expression of *STAT3*, *RHOA*, *GAS5*, and *LINC01116* genes in the cumulus cells of PCOS and control patients, as well as positive and negative pregnancy outcomes. Our goal is to assess the suitability of these genes as markers to predict oocyte competence and PCOS diagnosis.

## Materials and Methods

***Microarray data analysis***

The microarray data for GSE34526 was selected from the GEO database (http://www.ncbi.nlm.nih.gov/geo/) in the National Center of Biotechnology Information. This profile was based on human GCs obtained from ovarian aspirates of normal women and women with PCOS. A total of 7 cases of PCOS and 3 cases of control were profiled using the Affymetrix Human Genome U133 Plus 2.0 Array (HG-U133_Plus_2) platform. To evaluate microarray data quality, we conducted principal component analysis (PCA) using Python version 3.9.7 and Anaconda 4.10.3.

***Identification of differentially expressed genes (DEG)***

The GEO2R tool of the GEO database was used for the identification of DE genes; |Log2 fold change (log2FC)|≥1 and *P-value*<0.05 were regarded as cutoff criteria. The heatmap was plotted by ClustVis (http://biit.cs.ut.ee/clustvis/), and the volcano plot was prepared using the ggplot2 R package version 4.1.2.

***Function and pathway enrichment analysis***

In order to analyze the biological processes involved in the pathogenesis of PCOS, gene ontology (GO) enrichment and KEGG pathway analysis of differential genes were performed using g:Profiler (https://biit.cs.ut.ee/gprofiler). GO analysis included three components: BP (Biological Process), CC (Cellular Component), and MF (Molecular Function). *P*<0.05 was considered significantly different.

***PPI network and key genes***

To indicate cellular connections between cumulus cells and oocytes, we initially selected genes involved in the biological process of "cell communication.” To construct a protein-protein interaction (PPI) network, we uploaded certain genes to the STRING (https://string-db.org/) (Version 11.0) protein database. A threshold value was a score ≥ 0.4 (Medium confidence). Then, the PPI network was visualized using Cytoscape version 3.8.0. The Cytohubba plug-in in Cytoscape software was used for hub gene selection, and the first 10 hub genes were screened by degree.

***Identification of lncRNAs***

In the present study, various lncRNAs that regulate the first 10 hub genes were obtained from the LncRNA2Target database.

***Construction of the lncRNA-mRNA co-expression network and identification of hub genes and lncRNAs***

The network graph of the lncRNA-mRNA co-expression network was built and visualized through the “merge” function in Cytoscape (Version: 3.8.1). The 2 hub genes and 2 hub lncRNAs were calculated according to degree by the Cytohubba plugin in Cytoscape.

***Clinical subjects***

The participants included 51 controls and 32 PCOS women admitted to Hazrat-e Maryam Infertility Clinic (Shahid Beheshti Hospital) and Mushtaq Infertility Center in Isfahan from April 2020 to January 2021. All patients gave informed consent and were younger than 45 years of age. The study was approved by Isfahan University of Medical Sciences Ethics Committee (IR.MUI.MED.REC.1400.011) and carried out in compliance with the Helsinki Declaration.

The demographic and clinical features of the subjects, such as age, weight, prolactin (PRL) levels, vitamin D levels, and oocyte information, were collected.

***Cumulus cell collection***

Follicular fluid was obtained following a conventional GnRH antagonist ovarian stimulation protocol using transvaginal ultrasound-guided needle aspiration and cumulus-oocyte complex (COC) placed in a Synvitroflushing medium (Origio company, REF: 15760125A). A portion of the CCs surrounding a single oocyte was removed using two sharp needles under the loop microscope. Oocytes were put in Fert medium (ORIGIO.REF 83010010A), and cumulus cells were promptly transferred into a falcon tube containing phosphate-buffered saline (PBS). The falcon tube was centrifuged at 12000 g for 2 min, and the supernatant was discarded. Each patient’s cumulus cell mass was transported to a cryovial and immediately frozen at -80 °C.

The remaining CCs were extracted from the COC with hyaluronidase (SAGE IVF Inc. Hyaluronidase, REF art-4007), and sperm was injected into the egg. The ART procedure was carried out using either standard IVF, intracytoplasmic sperm injection (ICSI), or a combination of both. The condition of the oocytes was assessed 16 to 18 hr later for the presence or absence of pre-nuclei. The developmental status of the embryos was examined over the next few days to see if they had reached the two-cell or more stages, and then 2-3 embryos were transplanted into the mother’s uterus. The pregnancy results, including the β-hCG test, fertilization rate, and chemical pregnancy rate, were examined two weeks following the transfer. The clinical pregnancy was evaluated by ultrasound three weeks following the transfer to confirm the presence of a beating heart.

***Validation of the expression levels of candidate genes by qRT-PCR***

Total RNA was extracted using the BioFACT™ Total RNA Prep Kit (BIOFACT, Korea) according to the manufacturer’s instructions. One microgram of RNA was reverse transcribed using the BioFact™ 5X RT Pre-Mix kit (BIOFACT, Korea) with a random hexamer primer. qRT-PCR analysis was performed on the Mic qPCR instrument (Bio Molecular Systems, Australia) using gene-specific primers (Table 1) and RealQ Plus 2x Master Mix Green (Ampliqon, Denmark). Glyceraldehyde 3-phosphate dehydrogenase (*GAPDH*) was used as a housekeeping gene. The PCR amplification settings were 15 min at 95 °C, followed by 40 cycles of denaturation for 20 sec at 95 °C, annealing for 30 sec at 60 °C, and extension for 30 sec at 72 °C. All qRT-PCR experiments were performed in triplicate. The relative gene expression was measured using the 2^−ΔΔCt^ method as described previously (23).

***Statistical analysis***

SPSS version 26.0 and GraphPad Prism 9.3.1 software were used for data analysis and presented as the mean ± standard deviation (SD). The group differences between the two independent samples were examined by Student’s t-test and Mann-Whitney U-test. Analysis of covariance (ANCOVA) was used to rule out any age-related confounding factors. The Chi-squared tests were used to compare categorical variables, and the parametric variables were investigated using the Pearson correlation coefficient. A receiver operating characteristic (ROC) curve was employed to evaluate the diagnostic value, including the area under the curve (AUC), sensitivity, and specificity. *P*-values less than 0.05 were considered statistically significant.

## Results

***Identification of DEGs***

**Figure 1:**
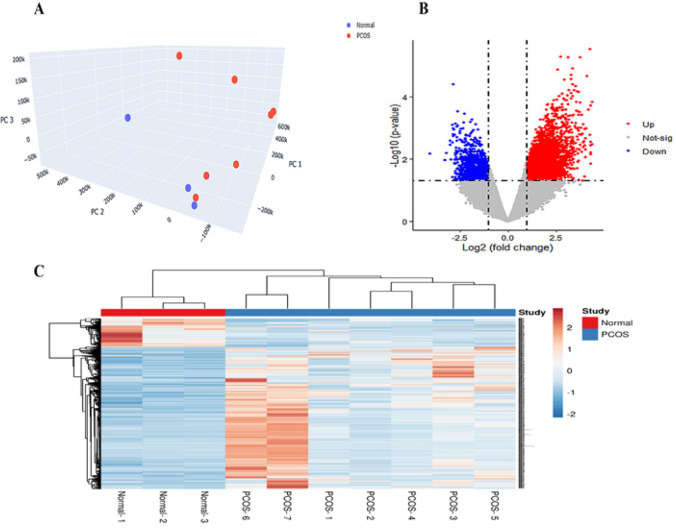
Microarray data analysis of 7 PCOS and 3 normal GCs samples

The gene expression profile GSE34526, which included 7 PCOS and 3 normal GCs samples, was obtained from the GEO database. The quality was assessed using PCA. The samples were separated according to the study groups in an unsupervised manner, indicating the acceptable quality of the dataset (Figure 1A). Based on the GEO2R analysis, 2721 DEGs, which included 1975 up-regulated and 746 down-regulated genes, were identified. As cut-off criteria, *P*-value 0.05 and |log2FC|≥ 1 were used. The relative expression levels of DEGs were shown in the volcano plot (Figure 1B). Additionally, the heat map of 1200 probes with the highest |log2FC| was shown in Figure 1C. The majority of genes in the PCOS samples were up-regulated as compared to those in the control samples. 

***Function and pathway enrichment of the DEGs***

**Figure 2:**
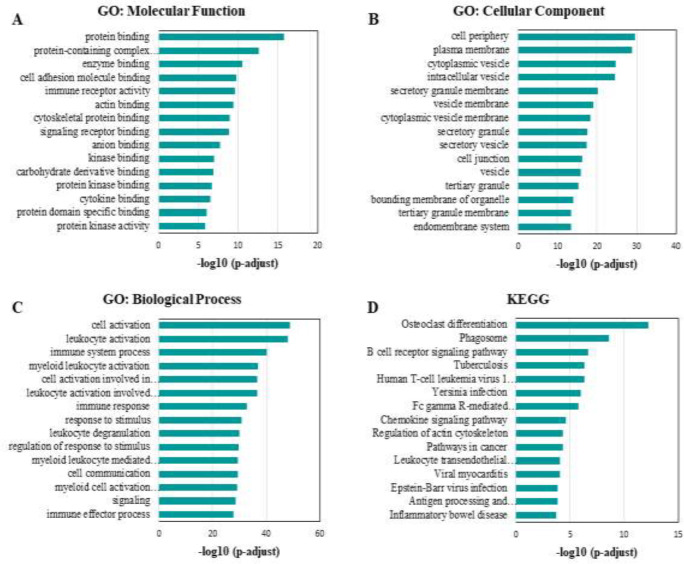
Function and pathway enrichment analysis of DEGs involved in the pathogenesis of PCOS

The g:Profiler online tool was used to perform GO enrichment and KEGG pathway analysis on 2721 DEGs. As shown in Figure 2, each part of the GO analysis shows the top 15 enrichment analysis results. Protein binding, enzyme binding, immune receptor activity, actin binding, and protein kinase activity are found in the molecular function (MF) section (Figure 2A). Cell periphery, plasma membrane, cytoplasmic vesicle, secretory granule, and cell junction are found in the cellular component (CC) (Figure 2B). Moreover, cell activation, leukocyte activation, immune system processes, response to stimulus, and cell communication are found in GO biological processes (BP) (Figure 2C). In particular, osteoclast differentiation, phagosome, B cell receptor signaling pathway, chemokine signaling pathway, and regulation of the actin cytoskeleton are mostly enriched in the KEGG pathway (Figure 2D). 

***PPI network construction and key genes***

**Figure 3:**
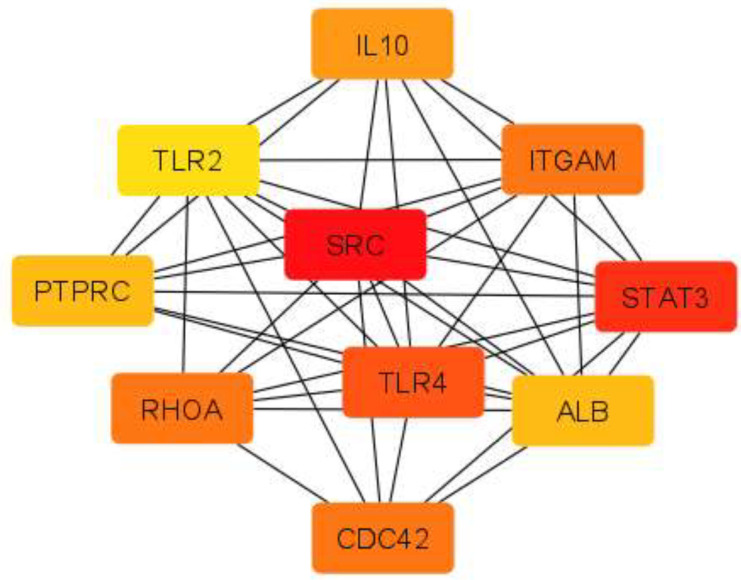
Hub gene network. Node color is related to its degree

The PPI network of selected genes involved in the biological process of “cell communication” was created to show cellular connections between cumulus cells and oocytes using STRING and imported into Cytoscape. The connection between each node was evaluated by adding an edge between them. The network was composed of 1173 nodes and 16478 edges. The top 10 highest-degree nodes in the PPI network are shown in Table 2 and Figure 3.

***Construction of the lncRNA-mRNA co-expression network and identification of hub genes and lncRNAs***

**Figure 4:**
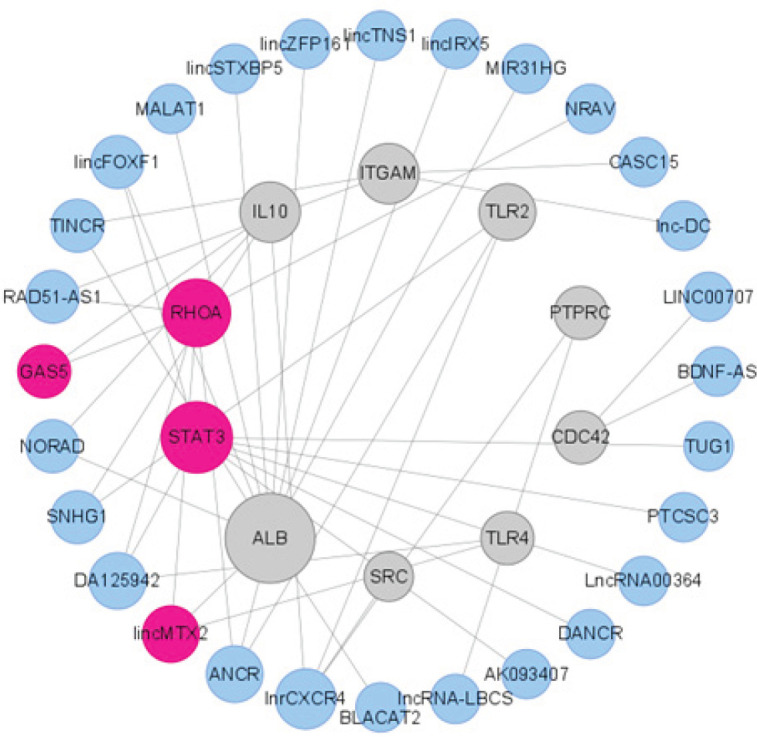
lncRNA-mRNA interaction network obtained from cytoscape

The lncRNA-mRNA interaction pairs of 10 hub genes were obtained from LncRNA2Target v2.0 (Table 3). Cytoscape software was used to construct an lncRNA–mRNA interaction network. In total, 43 lncRNA-mRNA pairs, including 28 lncRNAs and 10 mRNAs, were obtained. We analyzed the network, and 2 hub genes and 2 hub lncRNAs, including *STAT3*, *RHOA*, *GAS5,* and *lincMTX2 (LINC01116)*, were selected according to degree and previous studies (Figure 4) (Table 4).

***Clinical and demographic characteristics of PCOS and control subjects***

The clinical and biochemical characteristics of 51 controls and 32 PCOS patients who participated in this study are shown in Table 5. Results are presented as Mean ± SD. Patients with PCOS tend to be younger, with an average age of 30.88±5.06 years compared to 33.35±4.97 years for controls (*P=*0.041). The mean weight, PRL, and vitamin D level did not show significant differences between the two groups. The retrieved oocyte numbers per patient were higher in the PCOS group (13.97±8.85) compared to the control group (9.02±5.18) (*P=*0.005). The number of injected oocytes (*P=*0.014), GV oocytes (*P=*0.008), and 2PN embryos (*P=*0.049) was significantly fewer in the control group compared with the PCOS group. Although the fertilization rate was not significantly higher in the control group.

***Clinical and demographic characteristics of pregnant and non-pregnant groups***

There was no statistically significant difference between the pregnant (n = 42) and non-pregnant (n = 41) groups in age, weight, vitamin D, and PRL. The number of retrieved and GV oocytes was not significantly different between these two groups. Besides the quantity of injected oocytes and 2PN embryos, the fertilization rate was non-significantly lower in the pregnant group (Table 6).

***Type of treatment***

**Figure 5:**
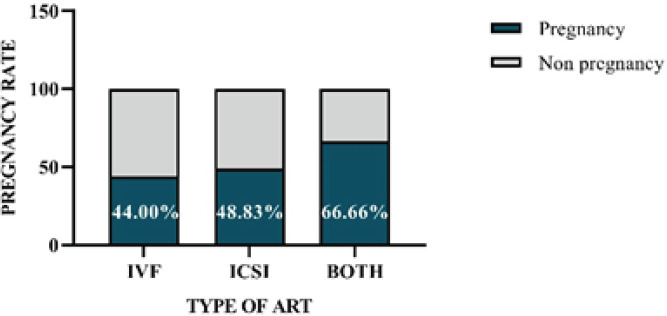
Comparison of the success rates of the various ART processes

83 women participated in this study, and they were divided into three groups. 25 patients (30.1%) were engaged in IVF, 43 patients (51.8%) were enrolled in ICSI, and 15 patients (18.1%) underwent both techniques. The pregnancy rate was slightly higher in the ICSI group than in the IVF group (48.83% vs 44.00%, respectively) and 66.66% in patients who used both procedures. But the difference did not reach significance by means of the chi-squared test (*P *= 0.361) (Figure 5).

***Validation of candidate genes by qRT-PCR***

To verify the candidate genes revealed by bioinformatics, four of the above-mentioned genes (*STAT3*, *RHOA*, *GAS5*, and* LINC01116*) on cumulus cells from all 32 PCOS patients and all 51 controls were evaluated by qRT-PCR. Also, the expression of these key genes was assessed in 42 pregnant and 41 non-pregnant women. Microarray analysis showed that *STAT3 *and *RHOA *were up-regulated DEGs in PCOS samples relative to normal samples.

**Figure 6:**
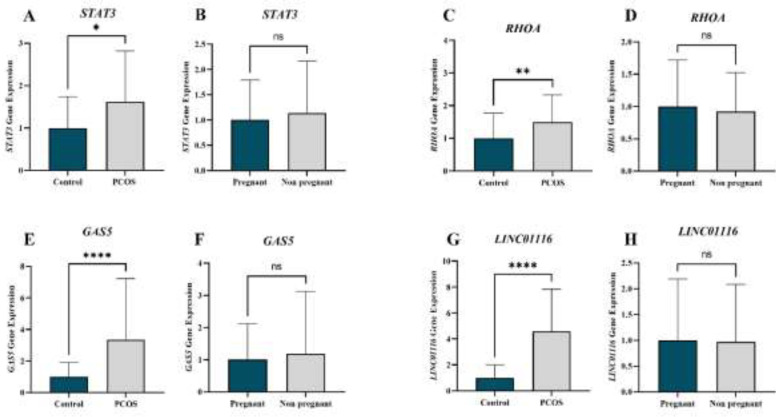
qRT-PCR analysis of the gene expression in human ovarian cumulus cells from PCOS and control and between pregnant and non-pregnant groups to verify the candidate genes revealed by bioinformatics, four of the above-mentioned genes (STAT3, RHOA, GAS5, and LINC01116

Without considering fertility results, when comparing the two groups of “control and PCOS”, increased levels of *STAT3 *(1.6-fold)*,* *RHOA *(1.4-fold), *GAS5 *(3.3-fold), and *LINC01116 *(4.5-fold) genes were found in PCOS patients, and the difference was significant (*STAT3 P=*0.0144) (*RHOA P=*0.001) (*GAS5 P<*0.0001) (*LINC01116 P<*0.0001) (Figure 6A, C, E, G). After adjusting for age, the difference in all four genes remained significant. 

As Figure 6 shows, in the comparison of “pregnant with non-pregnant”, regardless of PCOS and control groups, non-significantly increased expression levels of *STAT3 *and *GAS5 *were observed in the non-pregnant group compared to the pregnant group (Figure 6B, F). *RHOA *and *LINC01116* gene expression was slightly lower in the non-pregnant group compared to the pregnant ones, but the difference was not statistically significant (Figure 6D, H). The expression levels of genes showed no significant differences between these two groups after age was adjusted by ANCOVA.

***Association between candidate genes and fertilization rate***

**Figure 7:**
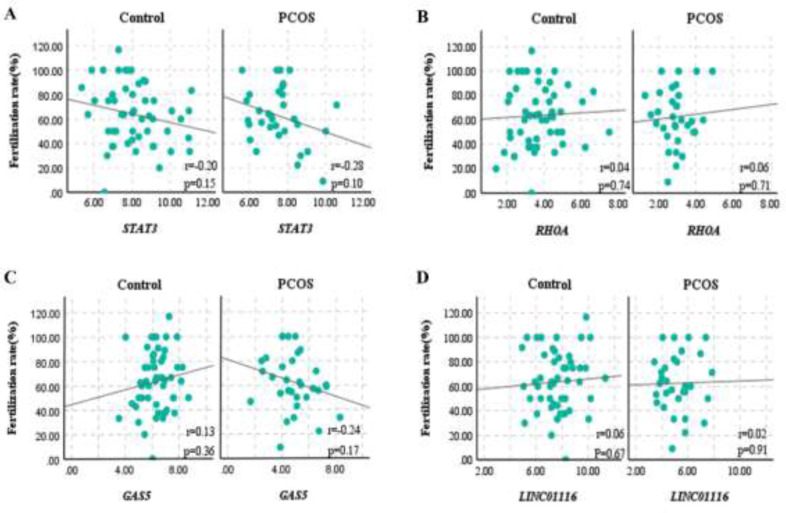
Scatter plots were used to depict the correlation between the gene expression of (A) *STAT3*, (B) *RHOA*, (C) *GAS5*, (D) *LINC01116* and fertilization rate in cumulus cells from control and PCOS patients

The correlation between candidate genes (*STAT3*,* RHOA*,* GAS5*, and* LINC01116*) and fertilization rate was analyzed. The results showed the expression of genes was not significantly associated with fertilization rate in the control and PCOS groups (*P>*0.05) (Figure 7).

***Correlation in gene expression pattern***

**Figure 8:**
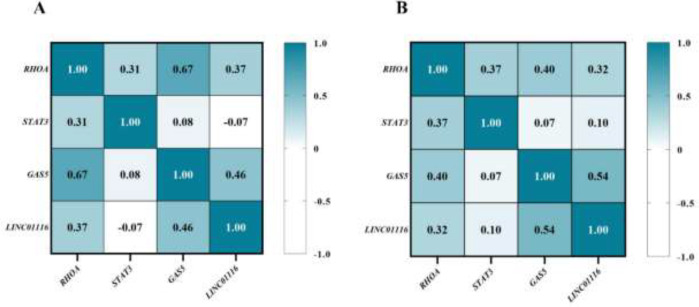
Correlation of gene expression in controls and PCOS patients

We used Pearson’s correlation to examine the relationship between candidate genes in controls and PCOS patients (Figure 8). In the control group, the results indicated a significant positive correlation of *RHOA *gene expression with* STAT3 *(r = 0.314, *P=*0.025), *GAS5* (r = 0.671, *P=*0.0001), and* LINC01116 *(r = 0.369,* P=*0.008), as well as a significant positive association between the expression of the *GAS5 *and *LINC01116* genes (r = 0.461, *P=*0.001) (Figure 8A).

In the PCOS patients, there was a significant positive correlation of *RHOA* gene expression with *GAS5 *(r = 0.403, *P=*0.022) and *STAT3 *(r = 0.369, *P=*0.038) and also a significant positive relationship between the *GAS5 *gene and *LINC01116 *gene expression (r = 0.540, *P=*0.001) (Figure 8B).

***Diagnostic values of gene expression for PCOS***

**Figure 9:**
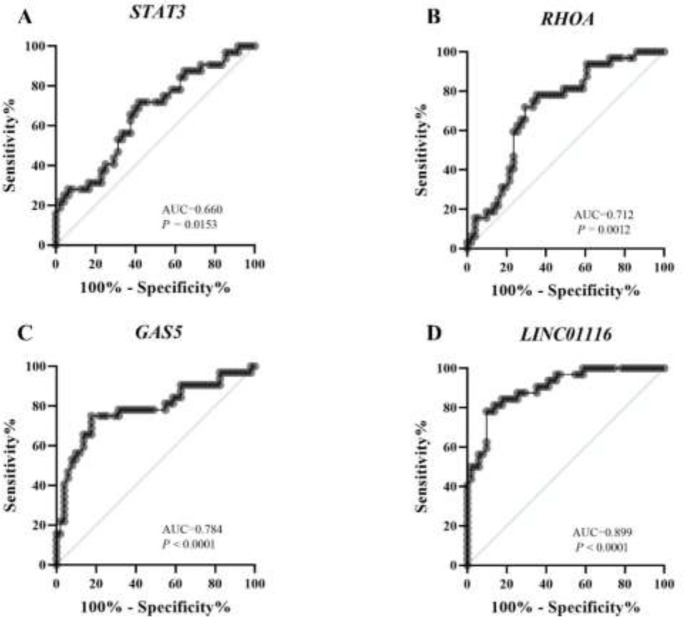
Receiver operating characteristics (ROC) curves of (A) *STAT3*, (B) *RHOA*, (C) *GAS5*, and (D) *LINC01116* in distinguishing PCOS and normal controls

ROC curves were produced, and AUC was determined to assess the diagnostic value of *STAT3, RHOA*, *GAS5*, and *LINC01116*. As shown in Figure 9, we found that the ROC curve of *STAT3 *indicated a significant distinguishing efficiency with an AUC value of 0.660 (95% CI: 0.53-0.78, *P=*0.0153) (Figure 9A), an AUC value of 0.712 for *RHOA *(95% CI: 0.60-0.82, *P=*0.0012) (Figure 9B), an AUC value of 0.784 for *GAS5* (95% CI: 0.67-0.89, *P<*0.0001) (Figure 9C), and an AUC value of 0.899 for *LINC01116 *(95% CI: 0.83-0.96, *P<*0.0001) (Figure 9D). We evaluated the discriminating effects of *STAT3, RHOA, GAS5,* and *LINC01116* at the cut-off values of 7.91, 3.29, 5.42, and 5.84, respectively, at which the greatest Youden’s index was determined as the optimal diagnostic point. As a consequence, the sensitivity and specificity of *STAT3,* *RHOA*,* GAS5, *and *LINC01116 *were 71.88% and 58.33%, 78.13% and 64.71%, 75% and 82.35%, and 78.13% and 90.2%, respectively. 

(A) Principal component analysis (PCA).The samples were separated according to the study groups. The red circle indicates the PCOS samples and the blue circle indicates the control samples. (B) Volcano plot of differential gene expression with log2 (fold change) as the abscissa and -log10 (*P*-value) as the ordinate. Red and blue dots denote significantly up-regulated and down-regulated genes, respectively, while grey dots indicate non-significant genes. (C) Heatmap of 1200 probes with the highest |log2FC| in the GSE34526 dataset. Each line represents one gene and each column represents one sample. Red color indicates a higher expression level; blue color indicates a lower expression level.

**Table 1:** The primer sequences of Real-Time PCR

**Gene name**	**Primer sequences**	**Product size (bp)**
*STAT3*	F: CAGCAGCTTGACACACGGTA R: AAACACCAAAGTGGCATGTGA	150
*RHOA*	F: GGAAAGCAGGTAGAGTTGGCT R:GGCTGTCGATGGAAAAACACAT	118
*GAS5*	F: CGACTCCTGTGAGGTATGGTG R: ATCCTTCCTTGGGGACACAAC	86
*LINC01116 *	F: CGCTTTGCTGAAGACGAGC R: ATATTGAACTGAGCGGGGCT	75
*GAPDH*	F: AAGCTCATTTCCTGGTATG R: CTTCCTCTTGTGCTCTTG	125

(A) Top 15 molecular functions of DEGs enriched in; (B) top 15 cellular components of DEGs enriched in; (C) top 15 biological processes of DEGs enriched in; (D) top 15 KEGG pathway analysis. DEGs, differentially expressed genes; KEGG, Kyoto Encyclopedia of Genes and Genomes.

DEGs: Differentially expressed genes; KEGG: Kyoto Encyclopedia of Genes and Genomes

**Table 2:** List of top ten hub genes according to degree

Gene name	Degree
*SRC*	253
*STAT3*	208
*TLR4*	206
*RHOA*	191
*ITGAM*	191
*CDC42*	191
*IL10*	187
*ALB*	186
*PTPRC*	186
*TLR2*	173

**Table 3:** The lncRNA-mRNA interaction pairs of 10 hub genes obtained from LncRNA2Target v2.0

**Gene target**	**Number of lncRNAs**	**lncRNA gene names**
*SRC*	1	*lnrCXCR4*
*STAT3*	7	*AK093407* *, * *DANCR* *, * *LncRNA00364* *, * *PTCSC3* *, * *TUG1* *, * *lincFOXF1* *, * *DA125942*
*TLR4*	2	*lincMTX2* *, * *DA125942*
*RHOA*	6	*lincMTX2* *, * *ANCR* *, * *DA125942* *, * *RAD51-AS1* *, * *GAS5* *, * *NRAV*
*ITGAM*	4	*lnc-DC* *, TINCR, RAD51-AS1, CASC15*
*CDC42*	2	*BDNF-AS* *, LINC00707*
*IL10*	4	*GAS5* *, lnrCXCR4, NORAD, SNHG1*
*ALB*	12	*MIR31HG* *, TINCR, lincFOXF1, lincIRX5, lincMTX2, lincTNS1, lincZFP161, lincSTXBP5, ANCR, MALAT1, NORAD, BLACAT2*
*PTPRC*	2	*lnrCXCR4* *, lncRNA-LBCS*
*TLR2*	3	*ANCR* *, lnrCXCR4, SNHG1*

There were 28 lncRNAs, 10 mRNAs, and 43 edges in the network. The size of the nodes indicates the degree of interconnectivity. Grey and blue nodes represent mRNAs and lncRNAs, respectively. Pink nodes indicate hub genes and lncRNAs.

**Table 4:** List of 2 hub genes and 2 hub lncRNAs, including STAT3, RHOA, GAS5, and lincMTX2

**Gene**	**Related lncRNAs**	**lncRNA**	**Related genes**
*STAT3*	*AK093407* *, DANCR, LncRNA00364, PTCSC3, TUG1, lincFOXF1, DA125942*	*lincMTX2	*TLR4* *, RHOA, ALB*
*RHOA*	*lincMTX2* *, ANCR, DA125942, RAD51-AS1, GAS5, NRAV*	*GAS5*	*RHOA* *, IL10*

*LINC01116

**Table 5:** The clinical and biochemical characteristics of 51 controls and 32 PCOS patients who participated in this study

**Parameter**	Control **N=51**	PCOS **N=32**	P*-value*
Age	33.35±4.97	30.88±5.06	*P* *=*0.041
Weight	67.29±7.93	66.69±7.63	*Ns*
Vitamin D (ng/mL)	29.32±11.25	32.57±15.25	*Ns*
PRL (ng/mL)	13.44±7.56	13.04±6.19	*Ns*
No. of Oocyte retrieve	9.02±5.18	13.97±8.85	*P=*0.005
No. of Oocytes injected	7.74±4.50	10.66±5.71	*P=*0.014
No. of GV oocytes	1.14±1.2	2.28±2.49	*P=*0.008
No. of 2PN embryos	4.72±3.02	6.65±4.24	*P=*0.049
Fertilization rate(%)	63.68±24.84	62.55±23.23	*Ns*

PRL: Prolactin, GV: Germinal vesicle, 2PN: 2 pronuclear, Ns: Non-significant. Germinal vesicle (GV): The egg has not begun meiosis yet, so it is considered immature. 2PN: A normally fertilized egg is called a 2PN for 2 pro-nuclei. Fertilization rate: No. oocytes with 2PN and 2PB / No. oocytes inseminated or injected×100%. Data presented as mean ± standard deviation (SD). Differences between groups were considered significant for *P*<0.05.

**Table 6:** Clinical and oocyte parameters in pregnant (n = 42) and non-pregnant (n = 41) groups

**Parameter**	Pregnant **N=42**	Non-pregnant **N=41**	*P-value*
Age	32.79±5.27	32.00±4.99	*Ns*
Weight	67.74±6.64	66.37±8.81	*Ns*
Vitamin D (ng/mL)	28.94±11.77	32.24±14.01	*Ns*
PRL (ng/mL)	13.30±6.38	13.26±7.69	*Ns*
No. of Oocyte retrieve	11.26±6.81	10.59±7.64	*Ns*
No. of Oocytes injected	8.85±4.90	8.87±5.49	*Ns*
No. of GV oocytes	1.62±2.18	1.54±1.53	*Ns*
No. of 2PN embryos	5.11±3.27	5.82±3.98	*Ns*
Fertilization rate (%)	59.07±21.75	67.52±25.85	*Ns*

PRL: Prolactin, GV: Germinal vesicle, 2PN: 2 pronuclear, Ns: Non-significant. Germinal vesicle (GV): The egg has not begun meiosis yet, so it is considered immature. 2PN: A normally fertilized egg is called a 2PN for 2 pro-nuclei. Fertilization rate: No. oocytes with 2PN and 2PB / No. oocytes inseminated or injected×100%. Data presented as mean ± standard deviation (SD). Differences between groups were considered significant for *P*<0.05.

25 patients (30.1%) were engaged in IVF, 43 patients (51.8%) were enrolled in ICSI, and 15 patients (18.1%) underwent both techniques. The difference was not found to be statistically significant.

(A) In the PCOS group, *STAT3 *gene expression was significantly higher than in the control group. (B) There was no significant difference in the expression of the *STAT3 *gene between the two groups. (C) In the PCOS group, a significantly higher level of *RHOA *was found compared to controls. (D) *RHOA *gene expression did not significantly differ between the positive and negative fertility groups. (E) GAS5 gene was up-regulated in PCOS patients compared to controls. (F) The expression of GAS5 in the group with positive fertility results was not significantly different from the group with negative fertility results. (G) *LINC01116* level was increased in the PCOS group compared to the control group. (H) There was no significant difference in *LINC01116* expression between the pregnant and non-pregnant groups. * *P*=0.01, ** *P*=0.001, **** *P*<0.0001. n=32 and 51 for PCOS and controls, respectively; n=42 and 41 for pregnant and non-pregnant groups, respectively.

PCOS: Polycystic ovary syndrome

The expression of genes was not significantly associated with fertilization rate in the two groups. Statistical analysis of the data was performed using the Pearson test.

PCOS: Polycystic ovary syndrome

(A) In the control group, *RHOA *was significantly and positively associated with GAS5 and *LINC01116*. (B) In PCOS patients, there was a significant positive correlation between *RHOA *gene expression with *GAS5* and *STAT3*. The Pearson test was used to statistically analyze the data.

A stronger diagnostic test results from a greater area under the curve

As a consequence, the sensitivity and specificity of STAT3, RHOA, GAS5, and LINC01116 were 71.88% and 58.33%, 78.13% and 64.71%, 75% and 82.35%, 78.13% and 90.2%, respectively.

## Discussion

Single embryo transfer is becoming more common in assisted reproduction in order to avoid adverse outcomes related to multiple pregnancies, and selecting embryos with high implantation capability for this purpose remains one of the crucial goals in the field of assisted reproduction (24). Gene expression profiling in CC could provide a non-invasive method for identifying the most competent oocytes and embryos (25). PCOS is the most common reproductive disorder affecting women in their fertile years (26).

In this study, to draw a two-layer network and discover candidate genes, we searched the GSE34526 dataset of PCOS from GEO and found 2721 DEGs. GO terms and KEGG pathway enrichment analysis of DEGs showed that these genes were primarily involved in cell activation, immune system processes, cell junction, regulation of the actin cytoskeleton, etc. Also, the PPI network of selected genes associated with the biological process of “cell communication” was analyzed, and the first 10 key genes were determined by degree. The lncRNA-mRNA associations were obtained, and a network was constructed that included 28 lncRNAs and 10 mRNAs. Two hub genes and 2 hub lncRNAs, including *STAT3*, *RHOA*, *GAS5,* and *LINC01116*, were selected according to degree.

 Shen *et al*. designed the study by downloading the microarray dataset GSE34526 from GEO. A total of 674 DEGs were retained, which were noticeably enriched in inflammation and immune-related processes. Eight modules were selected from the Reactome functional interaction (FI) network. Pathway enrichment analysis identified significant mechanisms, such as RhoA-related pathways, that may be involved in the pathogenesis of PCOS (27).

Based on research, regardless of the diagnosis of male factor infertility, applying ICSI was not related to improved post-fertilization reproductive and implantation rates, and chemical and clinical pregnancy rates were statistically higher in the IVF group as compared with the ICSI group (28, 29). This observation may have been made due to oocyte degeneration brought on by mechanical damage during the ICSI procedure (29, 30). Although we found that the pregnancy rate in the ICSI procedure (48.83%) was almost the same as the IVF method (44.00%), the difference did not reach significance by means of the chi-squared test. In one study, it was also mentioned that there were no variations in the pregnancy rate and live birth rate between these two processes (31).

One of the major goals of this research was to investigate biomarkers to predict oocyte competency and PCOS diagnosis. Our candidate genes were selected based on network data and have significant predictive value in the reproductive process, according to previous studies. Although only a few studies have compared the expression of these genes in PCOS and healthy people, as well as their implications for fertility and pregnancy.

The signal transducer and activator of transcription 3 (Stat3) protein is a transcription factor and a member of the Stat family. *STAT3* has a number of biological functions depending on the cell type, including cell proliferation, anti-apoptosis, and cell motility (32). The JAK/STAT3 pathway is one of the key mechanisms associated with PCOS pathogenesis (33). Microarray analysis revealed that *STAT3 *was an up-regulated DEG in PCOS, which was in accordance with the results of RT-PCR. But, in the study by Li *et al*., it was mentioned that the p-STAT3 level was significantly lower in the granulosa of PCOS compared with a control (34).

Based on observations, *STAT3 *is expressed in oocytes, granulosa, and theca cells within the ovary and plays multiple roles in the regulation of oogenesis, oocyte maturation, and quality (32, 35, 36). A study found that *STAT3* is polarized in the oocyte and may be implicated in the following development of the embryo (37). Surprisingly, a study on a mouse genetic model revealed that deletion of *STAT3* in oocytes had no influence on fertility and that it is not required for oocyte growth, maturation, fertilization, or subsequent embryonic development (38). In the present study, *STAT3* expression levels were not significantly increased in non-pregnant women compared to the pregnant group. The difference wouldn’t become substantial if the age factor was removed.

RhoA (Ras homolog gene family, member A) is a small GTPase protein that regulates several basic cellular activities, such as migration, adhesion, cytokinesis, survival, proliferation, and gene expression (39, 40). There was no notable study that compared the expression of the *RHOA* gene in PCOS and control groups in humans. Nevertheless, in the study done by Lui *et al*. on a mouse pancreatic cell line, they showed that by inhibiting *RHOA *simultaneously with F-actin organization disruption, insulin secretion was increased (41). Since modulation of insulin secretion is a key aspect of PCOS pathogenesis, regulation of *RHOA* activity and its signaling pathways may be associated with PCOS-related hormonal imbalances (27). In the current study, *RHOA* was significantly increased in the PCOS group compared to controls, which was consistent with the outcome of the microarray analysis.

A study reported that *RHOA* plays a critical role in oocyte maturation since it enhances actin assembly, spindle formation, and subsequent contractile ring development during polar body emission (42, 43). We found that the expression of *RHOA *in the non-pregnant group was lower than in the pregnant group. However, this decrease was not significant. The difference would not also become significant by removing the age parameter.

*GAS5* (growth arrest-specific RNA) is a long noncoding RNA that regulates cell growth, proliferation, apoptosis, and survival (44, 45). Lin *et al*. indicated that *GAS5* levels in serum were significantly lower in PCOS patients with insulin resistance (IR) than in PCOS patients without IR or non-PCOS individuals (44). PCOS is described by aberrant folliculogenesis, which shows up as more developing follicles at all stages of growth. *GAS5* is thought to contribute to the pathophysiology of PCOS and may play a part in granulosa cell proliferation (44). In contrast to the study mentioned above, Wang *et al*. revealed that *GAS5* was considerably up-regulated in the plasma of PCOS patients (46). Our data confirmed this result, and we found that *GAS5* levels in the cumulus cells of PCOS patients were significantly higher than in controls. *GAS5* expression was discovered in ovarian tissue, female germline stem cells, and oocytes and plays major regulatory functions in a variety of developmental processes and embryogenesis (47, 48). We found in this study that the expression of* GAS5* in the non-pregnant group was non-significantly higher than in the pregnant ones. By eliminating the age parameter, the difference in *GAS5 *expression across groups would not also become significant.

The long non-coding RNA *LINC01116* plays a role in the occurrence and progression of cancers such as epithelial ovarian carcinoma and breast cancer (49, 50). Evidence has revealed that *LINC01116* functions as a ceRNA to regulate the expression of target genes, cell proliferation, invasion, cell apoptosis, and tumorigenesis (49). There was no relevant investigation that compared the expression of this lncRNA between PCOS and healthy groups and looked into its connection to conception and pregnancy. Here, we found that *LINC01116 *gene expression was significantly increased in the PCOS group compared to the control group. Furthermore*, LINC01116* levels were slightly lower in the non-pregnant group compared to the pregnant group, but this difference was not statistically significant.

The relationship between candidate genes and the rate of fertilization has not been adequately studied. Our findings demonstrated that in the control and PCOS groups, expression of genes (*STAT3*,* RHOA*,* GAS5*, and* LINC01116*) did not significantly affect the fertilization rate (*P>*0.05).

Pairs of lncRNAs and their target genes were downloaded from the LncRNA2Target database, and we showed the interactions between two lncRNAs, *GAS5* and *LINC01116*, and *RHOA*. Accordingly, the analysis of our data showed that in the control group, *RHOA* was significantly and positively correlated with *GAS5* and *LINC01116* (r = 0.671 and r = 0.369, respectively). In the PCOS patients, there was a significant positive correlation of *RHOA* gene expression with *GAS5 *and *STAT3 *(r = 0.403 and r = 0.369, respectively). However, we were unable to identify a substantial investigation into the association between the expression levels of lncRNAs and *RHOA*. 

A study assessed the sensitivity and specificity of the differential expression patterns of serum *GAS5* in PCOS and non-PCOS individuals, with an AUC of 0.727 (44). In this study, the ROC curves of *STAT3, RHOA, GAS5, *and *LINC01116 *indicated a significant difference in efficiency. 

## Conclusion

In this study by bioinformatic analysis, four key genes were identified in the network. This research is the first to report the significant up-regulation of *STAT3, RHOA, GAS5, *and *LINC01116* in the cumulus cells of PCOS patients by qRT-PCR and may serve as a possible biomarker for PCOS. Nevertheless, there was no significant difference in their expression between fertile and infertile groups, and we could not report them as a diagnostic biomarker for oocyte competency. More studies with a large sample size should be done to confirm these preliminary results.

## Authors’ Contributions

B H collected the data, performed experiments, and wrote the manuscript; FS M designed the study; A A contributed to sample preparation; B H and M K analyzed the data and evaluated and interpreted the results; M K revised and supervised the article; SR M was involved in bioinformatics analysis. All authors approved the current version of the manuscript.

## Funding

This study was supported by the research fund of Isfahan University of Medical Sciences, Iran.

## Ethical Approval

All procedures performed in the present study were in accordance with the ethical standards of the Ethics Committee of Isfahan University of Medical Sciences (IR.MUI.MED.REC.1400.011) and the 1964 Helsinki Declaration and its later amendments.

## Conflicts of Interest

The authors declare that they have no conflicts of interest. 
